# The Impact of Transitioning From In-Person to Virtual Heart Transplantation Selection Committee Meetings: Observational Study

**DOI:** 10.2196/35490

**Published:** 2022-03-30

**Authors:** Rongzi Shan, Neha V Chandra, Jeffrey J Hsu, Stephanie Fraschilla, Melissa Moore, Abbas Ardehali, Ali Nsair, Rushi V Parikh

**Affiliations:** 1 Department of Medicine University of California, Los Angeles Los Angeles, CA United States; 2 Division of Cardiology University of California, Los Angeles Los Angeles, CA United States; 3 Division of Cardiac Surgery University of California, Los Angeles Los Angeles, CA United States

**Keywords:** telemedicine, transplantation, heart failure, physician, heart transplant, virtual meeting, interprofessional relations, health systems, selection committee

## Abstract

**Background:**

Heart transplant selection committee meetings have transitioned from in-person to remote video meetings during the COVID-19 pandemic, but how this impacts committee members and patient outcomes is unknown.

**Objective:**

The aim of this study is to determine the perceived impact of remote video transplant selection meetings on usability and patient care and to measure patient selection outcomes during the transition period from in-person to virtual meetings.

**Methods:**

A 35-item anonymous survey was developed and distributed electronically to the heart transplant selection committee. We reviewed medical records to compare the outcomes of patients presented at in-person meetings (January-March 2020) to those presented during video meetings (March-June 2020).

**Results:**

Among 83 committee members queried, 50 were regular attendees. Of the 50 regular attendees, 24 (48%) were physicians and 26 (52%) were nonphysicians, including nurses, social workers, and coordinators; 46 responses were received, 23 (50%) from physicians and 23 (50%) from nonphysicians, with 41 responses fully completed. Overall, respondents were satisfied with the videoconference format and felt that video meetings did not impact patient care and were an acceptable alternative to in-person meetings. However, 54% (22/41) preferred in-person meetings, with 71% (15/21) of nonphysicians preferring in-person meetings compared to only 35% (7/20) of physicians (*P*=.02). Of the 46 new patient evaluations presented, there was a statistically nonsignificant trend toward fewer patients initially declined at video meetings compared with in-person meetings (6/24, 25% compared to 10/22, 45%; *P*=.32).

**Conclusions:**

The transition from in-person to video heart transplant selection committee meetings was well-received and did not appear to affect committee members’ perceived ability to deliver patient care. Patient selection outcomes were similar between meeting modalities.

## Introduction

In 2020, 3658 heart transplants were performed in the United States, with an additional 3576 candidates remaining on the waiting list, reflecting the national scarcity of donor hearts and the challenging decisions made by transplant centers during organ allocation [1]. Thus, transplantation selection committees are typically large multidisciplinary groups, including physician and nonphysician members, that complete comprehensive patient evaluations to determine transplant listing eligibility [2].

The COVID-19 pandemic transformed clinical and administrative practices in heart transplantation. One fundamental change was the transition of heart transplant selection committee meetings from in-person to remote videoconference meetings to maintain social distancing requirements. Current data explore digital health in the remote monitoring of patients with heart failure and patients receiving heart transplants, but the impact of telemedicine on heart transplantation selection committee meetings has not been studied as extensively [3,4]. Furthermore, there is limited data on provider satisfaction with telemedicine and virtual collaborations among providers [5]. Thus, this report aims to (1) understand how the transition of heart transplant selection committee meetings from in-person to remote videoconference has affected committee members and the perceived impact on patient care and (2) determine the impact of in-person compared to remote meetings on patient selection outcomes.

## Methods

### Recruitment

A 35-item anonymous survey was developed and distributed electronically to individuals on the adult heart transplantation selection committee roster at a single tertiary care academic hospital in May 2020. The survey was adapted from the validated Telehealth Usability Questionnaire [6] and developed in reference to the institution’s preferred videoconferencing system, Zoom (Zoom Video Communications Inc). Survey items included multiple-choice, Likert scale, and free-text responses and are available in Multimedia Appendix 1.

Selection committee meeting notes and electronic medical records were reviewed to obtain patient demographic characteristics, transplant listing status, and meeting outcomes. Data on the duration of meetings were not collected. Patients were included in the analysis if they were presented as a new evaluation to the adult heart transplant selection committee between January 3, 2020, and June 5, 2020, and had not already been chosen to receive a transplant by the start of the meeting. For patients whose decision was deferred, meaning they did not receive a decision at the initial meeting and were presented again at later meetings, their clinical course was followed beyond the original meeting to record the final decision and time to decision. 

### Ethics Approval

Informed consent was obtained from the committee members surveyed. The study protocol was approved by the University of California, Los Angeles Institutional Review Board (IRB#21-000084).

### Statistical Analysis

Quantitative descriptive analyses were performed, including subgroup analyses stratified by physician and nonphysician respondents. Likert scale responses were analyzed as continuous variables and averaged. To test for differences between groups, *t* tests were used for normally distributed variables. For the patient selection outcomes data, we measured the proportion of patients who were accepted, declined, or received a deferred decision, as well as the time to decision, using the Wilcoxon rank-sum test for skewed variables and chi-square tests for categorical variables. Statistical analyses were performed using Stata (version 15.1; StataCorp). *P* values <.05 were considered statistically significant.

## Results

### Survey Data

The heart transplant selection committee included 83 members, 50 of whom were regular attendees; of the 50 regular attendees, 24 (48%) were physicians and 26 (52%) were nonphysicians. Overall, 46 anonymous responses were submitted and included in the participant demographic analysis; however, 1 physician response was excluded from additional analyses since the respondent had not attended any videoconference meetings in the preceding 6 months (by self-report). Of the 46 survey respondents, 23 (50%) were physicians and 23 (50%) were nonphysicians. Physicians from the departments of medicine, surgery, and anesthesia were represented, with cardiologists comprising the majority (11/23, 48%) of physician respondents (Table 1). Nonphysicians included cardiomyopathy nurses, pharmacists, transplant coordinators, social workers, and administrators. At the time of the survey, 91% (42/46) of respondents had attended more than one video meeting, and complete responses were received from 41 participants.

Overall, both physician and nonphysician respondents were satisfied with video meetings regarding ease of use, interface quality, and interaction ability. Respondents agreed that they could contribute effectively to the meeting and achieve their clinical and administrative goals through videoconference. The predominant positive attributes of in-person meetings were communication and clinical decision-making, while location was the predominant negative. The predominant positive attributes of video meetings were multitasking, technology integration, and location convenience, while communication was the predominant negative (Table 2).

**Table 1:** Distribution of multidisciplinary committee member survey respondents by committee member subtype and physician subtype.

Selection committee member types			Value, n (%)
**Professional role (N=46)**			
	Cardiomyopathy registered nurse/nurse practitioner	4 (9)	
	Transplant and pretransplant coordinator	6 (13)	
	Ventricular assist device coordinator	3 (7)	
	Physician	23 (50)	
	Pharmacist	1 (2)	
	Dentist	1 (2)	
	Quality assurance professional	2 (4)	
	Financial counselor/coordinator	1 (2)	
	Social worker	2 (4)	
	Other	3 (7)	
**Medical specialty^a^ (n=23)**			
	Cardiology	11 (48)	
	Nephrology	1 (4)	
	Infectious diseases	5 (22)	
	Pulmonary	1 (4)	
	Anesthesiology	3 (13)	
	Surgery	2 (9)	

^a^These data were collected from physician respondents.

**Table 2:** The proportion of respondents that identified each meeting attribute as a positive or negative aspect of in-person or video meetings (N=45).

Meeting type and attribute			Identified as positive, n (%)		Identified as negative, n (%)
**In person meeting**					
	Location	11 (24)		18 (40)	
	Workflow	14 (31)		7 (16)	
	Communication	37 (82)		1 (2)	
	Multitasking	12 (27)		12 (27)	
	Clinical decision-making	28 (62)		1 (2)	
	Technology	5 (11)		13 (29)	
**Video meeting**					
	Location	28 (62)		1 (2)	
	Workflow	14 (31)		5 (11)	
	Communication	11 (24)		26 (58)	
	Multitasking	34 (76)		1 (2)	
	Clinical decision-making	12 (27)		12 (27)	
	Technology	24 (53)		5 (11)	

Concerns with communication included the inability to see attendees (ie, from video cameras being turned off), audio interruptions, and barriers to communication flow. Compared to nonphysicians, more physicians cited workflow as a positive aspect of video meetings (11/22, 50% of physicians compared to 3/23, 13% of nonphysicians) and a negative aspect of in-person meetings (6/22, 27% of physicians compared to 1/23, 4% of nonphysicians). Additionally, physicians more frequently identified clinical decision-making as a negative aspect of video meetings (8/22, 36% of physicians compared to 4/23, 17% of nonphysicians).

Overall, committee members did not feel that video meetings impacted their ability to engage in patient care, such as by clarifying clinical questions, creating management plans, and determining or updating transplant listing status. However, compared to nonphysicians, physicians had consistently lower mean Likert scale scores for questions regarding patient care improvement with video meetings. Physicians did not agree that videoconference meetings improved their ability to clarify clinical questions, while nonphysicians agreed (2.79 mean physician score compared to 3.48 mean nonphysician score; *P*=.03). Physician responses were neutral or in agreement for other patient care tasks, such as creating management plans and determining or updating transplant listing status.

Respondents agreed that videoconferencing was an acceptable alternative to in-person meetings (3.98 mean Likert score) but did not agree that the 2 meeting formats were equivalent (2.98 mean Likert score). Among all respondents, 54% (22/41) preferred the in-person meeting format for future selection committee meetings. When stratified by committee member subtype, 71% (15/21) of nonphysicians preferred in-person meetings compared with 35% (7/20) of physicians (*P*=.02).

### Patient Selection Outcomes

Of the 46 patients presented as new evaluations at heart transplant selection committee meetings from January to June 2020, the mean age was 54 (SD 2.1) years, 65% (n=30) were male, and 80% (n=37) were under consideration for single organ transplant (n=9, 20% were under consideration for multiple organ transplants). These characteristics were similar between in-person and video meetings. A total of 22 patients were presented during the in-person meeting phase (January-March 2020) and 24 patients were presented during the videoconferencing phase (March-June 2020).

As shown in Table 3, there was a numerical but statistically nonsignificant trend toward fewer patients initially declined at video meetings compared with in-person meetings (6/24, 25% compared to 10/22, 45%; *P*=.32), while more video patients were ultimately approved (16/24, 67% compared to 12/22, 55%; *P*=.40). Among the patients whose decision was deferred at the initial meeting, the median time to a final decision was 37 (IQR 21-124) days for in-person and 68 (IQR 27-97; *P*=.90) days for video meetings.

**Table 3:** Patient outcomes for both in-person and video selection committee meetings, N=46

Patient outcomes		In-person meetings (n=22)	Video meetings (n=24)	*P* value
**Initial decision at time of meeting, n (%)**				
	Declined	10 (45)	6 (25)	.32^a^
	Approved	5 (23)	9 (38)	
	Decision deferred	7 (32)	9 (38)	
**Final decision, n (%)**				
	Declined	10 (45)	8 (33)	.40^a^
	Approved	12 (55)	16 (67)	
**Time to final decision**				
	No delay in decision, n (%)	16 (73)	16 (67)	.66^a^
	Time to decision^b^ in days, median (IQR)	37 (21-124)	68 (27-97)	.90^c^

^a^*P* value obtained from the chi-square test.

^b^Among patients with a delayed decision (6 for in-person meetings and 8 for video meetings).

^c^*P* value obtained from the Wilcoxon rank-sum test.

## Discussion

### Principal Findings

The transition of heart transplant selection committee meetings from in-person to videoconference during the COVID-19 pandemic was well-received by committee members, though a higher proportion of physician members preferred video meetings than nonphysician members. Committee members perceived that video meetings did not impact patient care delivery. Patient selection outcomes for new patient evaluations did not significantly differ between the in-person and video meeting phases.

### Comparison to Prior Work

This study was unique in focusing on a digital experience among heart transplant professionals, while prior work in the area of telemedicine and heart transplantation focused on patient-facing interventions [7,8]. A prior qualitative study on liver transplant selection committee members found that the main barriers to decision-making included a lack of written policies, difficulty maintaining the balance between advocating for a patient and promoting organ stewardship, inconsistent attendance, and lack of efficiency [2]. In this study, physicians preferred video selection committee meetings, while nonphysician members preferred in-person meetings, which may be influenced by physicians’ perception of an improvement in efficiency when using videoconference. However, despite their preference for video meetings, physicians had lower agreement regarding whether video meetings improved patient care and more frequently cited clinical decision-making as a negative attribute of video meetings. In contrast, nonphysicians preferred in-person meetings, yet had higher agreement for questions relating to improvement in patient care delivery with video meetings. Collectively, these data highlight the nuanced nature of multidisciplinary heart transplant selection committee meetings and the perceived trade-offs of different meeting formats.

We observed numerical but statistically nonsignificant trends toward fewer immediately declined patients and more patients approved after some delay in the video meeting group. Given that respondents thought communication and clinical decision-making were easier with in-person meetings, a potential explanation may be a tendency to delay difficult clinical decisions in video meetings in favor of additional evaluation or monitoring over time.

### Strengths and Limitations

This was a small, retrospective, single-center study, so findings should be considered hypothesis-generating. The quantitative survey methods allowed for measurement of the perceived impact of virtual meetings with comparison to observed patient outcomes. The voluntary aspect of the survey may have introduced selection bias, though the anonymous nature limited response bias. The observed response rate (46/83, 55%) was low; however, the number of respondents was similar to the number of regular meeting attendees (46 compared to 50), though survey anonymity precluded our ability to identify if respondents were indeed regular attendees. This survey evaluated committee members soon after the change in meeting format to videoconferencing; these initial preferences may have evolved over time. Finally, patient selection outcomes may have been affected by unmeasured confounders such as the unpredictable and evolving impact of the COVID-19 pandemic on programmatic transplant policies over time.

### Future Directions

These observations warrant further investigation in larger studies. A sample size of 275 patients in each group (in-person and video meetings) would be required to detect the proportions observed in this pilot study in an adequately powered trial (80% power at a 2-tailed α of .05). Future studies should assess team interaction in virtual and in-person meetings, such as engagement with colleagues and ability to advocate for patients, as well as the impact on efficiency and attendance. Additionally, registry data to assess outcomes across multiple transplant centers could be incorporated.

### Conclusions

The videoconferencing format for heart transplant selection committee meetings was generally well-received by the multidisciplinary members, though physicians reported a greater preference for video meetings compared to nonphysicians. Overall, video meetings do not affect committee members’ perception of their ability to deliver patient care, which is corroborated by similar patient selection outcomes across both meeting modalities. Additional studies are needed to evaluate the impact of virtual meetings on care delivery systems and transplant-related patient outcomes.

## Appendix

Multimedia Appendix 1 Survey items (excludes demographic and free-text items).

## Data Availability

Deidentified data can be accessed by contacting the corresponding author.
