# Supplementary material for: The Impact of Transitioning From In-Person to Virtual Heart Transplantation Selection Committee Meetings: Observational Study
Source: JMIR Cardio. 2022 Mar 30;6(1):e35490. doi: 10.2196/35490 (PMC9008536; doi:10.2196/35490)
Supplement: Multimedia Appendix 1 [file cardio_v6i1e35490_app1.docx]

**Supplementary Table 1**. Survey Items (excludes demographic and free-text items)

| **Question** | **Attribute** |
| --- | --- |
| It is simple to use the video conference system (Zoom Video Communications). | Ease of use |
| I like using the current video conference system (Zoom Video Communications). | Interface quality |
| Using the video conference system: - I can hear the other meeting attendees. | Interaction quality |
| Using the video conference system: - I can see the other meeting attendees. | Interaction quality |
| Using the video conference system: - I can see the patient list being discussed. | Interaction quality |
| Using the video conference system: - I can easily talk to the other meeting attendees. | Interaction quality |
| Using the video conference system: - I feel comfortable communicating with the meeting attendees. | Interaction quality |
| Using the video conference system: - I can contribute to the discussion of a patient in a timely manner. | Interaction quality |
| Video conference allows me to: - Achieve my clinical and patient care goals for a transplant committee meeting. | Usefulness |
| Video conference allows me to: - Achieve my administrative goals for a transplant committee meeting. | Usefulness |
| Video conference allows me to: - Contribute effectively to the transplant committee meeting. | Usefulness |
| Video conference allows me to: - Save time traveling to a physical meeting location. | Usefulness |
| I think the video conference meeting is the same as an in-person meeting. | Reliability |
| Video conference is an acceptable way to conduct adult heart transplant selection committee meetings. | Satisfaction & Future Use |
| Overall, I am satisfied with video conference-based adult heart transplant selection committee meetings. | Satisfaction & Future Use |
| If given the option, for the next transplant committee meeting, I would choose the following meeting format: | Satisfaction & Future Use |
| How frequently do you access a patient's medical records (EMR or paper) during: - In-person transplant committee meetings? | Participant Behavior |
| How frequently do you access a patient's medical records (EMR or paper) during: - Video conference transplant committee meetings? | Participant Behavior |
| Compared to in-person meetings, video conference transplant committee meetings improve real-time decision-making about the following aspects of patient care: - Clarification of clinical questions or discrepancies | Clinical Care Outcomes |
| Compared to in-person meetings, video conference transplant committee meetings improve real-time decision-making about the following aspects of patient care: - Patient management plan | Clinical Care Outcomes |
| Compared to in-person meetings, video conference transplant committee meetings improve real-time decision-making about the following aspects of patient care: - Transplant listing determination | Clinical Care Outcomes |
| Compared to in-person meetings, video conference transplant committee meetings improve real-time decision-making about the following aspects of patient care: - Changes or updates to transplant listing status | Clinical Care Outcomes |
| Compared to in-person meetings, video conference meetings lack a tangible or intangible aspect that ultimately impacts patient care. | Clinical Care Outcomes |
| Please indicate the positive and negative aspects of **in-person meetings.**Select multiple options if applicable.  Options: Location, Workflow, Communication, Multitasking, Clinical decision making, Technology | Positive and Negative Attributes |
| Please indicate the positive and negative aspects of **video meetings.**Select multiple options if applicable.  Options: Location, Workflow, Communication, Multitasking, Clinical decision making, Technology | Positive and Negative Attributes |
